# Targeted Delivery of Plasminogen Activators for Thrombolytic Therapy: An Integrative Evaluation

**DOI:** 10.3390/molecules24183407

**Published:** 2019-09-19

**Authors:** Yunn-Hwa Ma, Chih-Hsin Liu, Yueh Liang, Jyh-Ping Chen, Tony Wu

**Affiliations:** 1 Department of Physiology and Pharmacology, College of Medicine, Chang Gung University, Taoyuan 33302, Taiwanmat90011@yahoo.com.tw (Y.L.); 2 Department of Neurology, Chang Gung Memorial Hospital, Taoyuan 33305, Taiwan; tonywu@adm.cgmh.org.tw; 3 Graduate Institute of Biomedical Sciences, Chang Gung University, Taoyuan 33302, Taiwan; 4 Department of Chemical and Materials Engineering, College of Engineering, Chang Gung University, Taoyuan 33302, Taiwan; jpchen@mail.cgu.edu.tw

**Keywords:** plasminogen activators, nanoparticles, drug delivery, thrombolysis

## Abstract

In thrombolytic therapy, plasminogen activators (PAs) are still the only group of drug approved to induce thrombolysis, and therefore, critical for treatment of arterial thromboembolism, such as stroke, in the acute phase. Functionalized nanocomposites have attracted great attention in achieving target thrombolysis due to favorable characteristics associated with the size, surface properties and targeting effects. Many PA-conjugated nanocomposites have been prepared and characterized, and some of them has been demonstrated with therapeutic efficacy in animal models. To facilitate future translation, this paper reviews recent progress of this area, especially focus on how to achieve reproducible thrombolysis efficacy *in vivo*.

## 1. Problems in Treatment of Thromboembolic Diseases

Intravascular thromboembolic events, including myocardial infarction, ischemic stroke, peripheral arterial thrombosis, deep vein thrombosis, and pulmonary embolism, are common and potentially life-, organ-, and limb-threatening vascular diseases. These events are commonly associated with ischemia and tissue necrosis, symptoms with high risk of mortality. Although intravascular procedure for removal of the clot provides a beneficial outcome for certain patients with thromboembolism, plasminogen activators (PAs) are still the only group of drugs recommended for acute treatment of thromboembolism in the central arteries in clinical practice [1,2]. As a group of enzymes from a variety of sources, PAs convert plasminogen in the thrombus to plasmin that prompts degradation of fibrin, which is the major structural component in the clot. PA-induced fibrin degradation and blood flow restoration is still the only strategy available in clinical setting without requirement of advanced surgical intervention. Tissue-type plasminogen activator (tPA) is an endogenous protein produced by vascular endothelium, which plays an important role in the physiological regulation of hemostasis and thrombolysis. Intravenous (*i.v.*) delivery of recombinant form of tPA (rtPA) remains the only treatment approved for acute ischemic stroke. Due to the presence of endogenous inhibitors in circulation, traditional rtPA (alteplase) exerts a short half-life of 4–6 min [3]. This requires a large *i.v.* dose followed by continuous infusion to achieve therapeutic efficacy, causing risks of inducing hemorrhagic complication. Newly developed rtPA, such as reteplase or tenecteplase, with depletion or mutation of certain amino acids, respectively, exerts longer half-lives and thus simplifies dosing schemes [4]. However, the comparable efficacy and safety are still associated with hemorrhagic complication that may be potentially overcome by targeted delivery strategy using nanocarriers [5].

Many rtPA nanocomposites have been prepared and tested for delivery of rtPA; however, none of them has passed clinical trial. Recently, advantages and disadvantages of different designs of rtPA nanocomposites have been extensively reviewed [5,6]. The purpose of this article is to focus on details of integrative evaluation of rtPA nanocomposites in test tubes (*in vitro*) and in animal models (*in vivo*), in order to facilitate translation in the future. Therefore, only studies with animal models that demonstrate results with statistical significance are included.

## 2. Design and Preparation of rtPA Nanocomposites

Targeted delivery of PAs using nanocarriers has been demonstrated to be a feasible strategy in animal models. A variety of carrier systems have been tested for delivery of PAs, including liposomes, polymer-based nanoparticles, microbubbles, and magnetic nanoparticles (MNPs) etc., with different advantages and disadvantages [5,6]. It is generally considered that lipid-bilayer structured liposomes [5,7] or cell-derived carriers [8] are most biocompatible, whereas the biocompatibility of polymeric nanoparticles and MNPs may depend on the characteristics of the polymers as the coating materials [9]. In addition to biocompatibility, the coating materials also provide better colloidal stability and functional groups for immobilization of targeting ligands or PAs. The iron oxide (Fe_3_O_4_ or γ-Fe_2_O_3_) core of MNPs with superparamagnetic properties enables magnetic separation and magnetic capture in a drug delivery system [9].

Recently, the design of the carrier system adopting more than one concept facilitates optimization of the physical and/or chemical characteristics of the carriers to allow controlled release of rtPA. For instance, echogenic liposomes was prepared by incorporating gas bubbles and rtPA in the aqueous compartment of the lipid-bilayer structure of liposomes, allowing rtPA release controlled by ultrasound [10]. By incorporating MNPs and rtPA into thermal sensitive liposomes, the magnetoliposomes can be magnetically guided *in vivo* with rtPA release controlled by elevated temperature at the target site [11].

**Figure 1:**
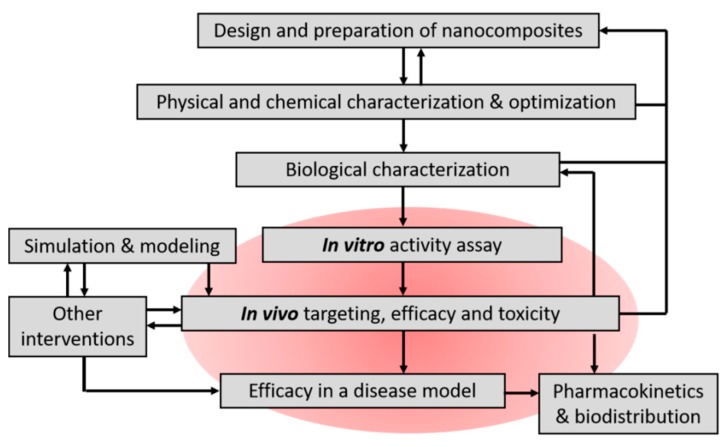
Schematic diagram of proposed steps in development of therapeutic nanocomposites for target thrombolysis. Arrows indicate sequences or feedback consideration in the development. This review focuses on the pharmacological evaluation of the nanocomposites, as included in the red area.

This area of research requires cross-disciplinary corporation to handle material preparation, characterization, and *in vitro*/*in vivo* activity assay. A flow chart in Figure 1 suggests how magnetic nanocomposites for thrombolysis and other therapeutic purposes may be prepared and tested. At certain steps, experimental results may feedback to previous steps to further optimize the preparation, such as the physical/chemical and biological characteristics of the nanocomposites, and drug activity tested *in vitro*. Since PAs are protein drug and the enzyme activity may be compromised during manufacturing, it is essential to check the enzyme activity at critical steps.

### 2.1. Immobilized PAs

**Figure 2:**
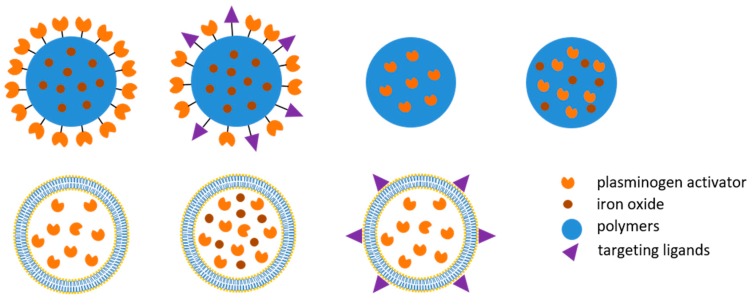
Illustration of thrombolytic nanocomposites demonstrating pharmacological efficacy in a disease model *in vivo*. Both immobilized and protected plasminogen activator in the polymeric (upper panel) and liposomal (lower panel) nanocomposites can be used.

The functionalization of nanoparticles is a critical process, which defines the properties and stability of the nanocomposites. Covalent immobilization of rtPA on the surface of the nanocarriers is often achieved by carbodiimide-mediated amide bond formation, potentially jeopardizing the enzyme activity of rtPA [12]. However, covalent immobilization permits a stable and reproducible preparation that improves the reactivity compared to the adsorbed rtPA preparation [13]. It has been shown that increased loading efficiency may overcome the reduced activity of rtPA caused by immobilization [14]. It has also been demonstrated that urokinase, another PA, can be adsorbed onto heparin-coated iron oxide aggregates and act as a thrombolytic agent *in vitro* and *in vivo* [15]. The immobilization allows calculation of the equivalent PA concentration or dose in the following evaluation. Nanocomposites with immobilized PAs on the surface (Figure 2) permit PAs to act directly on the thrombus that lodges in the arteries. Although a quite wide range of rtPA mass/carrier may all induce thrombolysis *in vitro*, only high enough of PA mass/carrier may induce effective thrombolysis *in vivo* [14].

### 2.2. Protected PAs

The major advantage of protected or encapsulated PAs is reduced inactivation by endogenous inhibitors before reaching the target. Activity of immobilized PAs may be compromised due to steric hindrance; whereas for protected PAs, the local concentration at the target is greatly dependent on the release kinetics or the strategy of controlled release. There have been liposomal preparations [11,16,17,18,19,20] and polymeric nanocomposite [21,22] developed and achieved target thrombolysis *in vivo*, as shown in Figure 2. The protection may extend its half-life [20], allowing PAs to be administered from a remote site in circulation such as vein [11,16,17,18]. However, the challenges of encapsulated rtPA remain to control the timing or location of the release of the load. One useful strategy is to use targeting ligand that allows presumably better association of the liposome with fibrin [16] or activated platelets [17,20] in the thrombus, resulting in higher PA concentration at the target for longer time. In the case of thermosensitive magnetoliposome encapsulating rtPA, focal hyperthermia may induce release of rtPA at the site of thrombus and induce restore of blood flow [11]; whereas ultrasound may trigger release of rtPA from an echogenic liposome preparation to enhance thrombolysis [19]. Pawlowski et al. designed liposomal nanocomposites that respond to secreted phospholipase A2 from activated platelets and unload the encapsulated PA; such nanocomposites allows release of the thrombolytic drug without additional intervention after reaching the target site [23].

## 3. Targeting Strategy

For target thrombolysis with the thrombus located in the vessels, PA nanocomposites have to overcome hydrodynamic force to act on the plasminogen located inside the thrombus. For magnetic nanocomposites, the targeting effects can be achieved by magnetic guiding under magnetic field at the peri-vascular site; however, targeting effects may be achieved for all PA nanocomposites using a ligand that specifically binds to the molecule target on or inside the thrombus.

### 3.1. Magnetic Targeting

**Figure 3:**
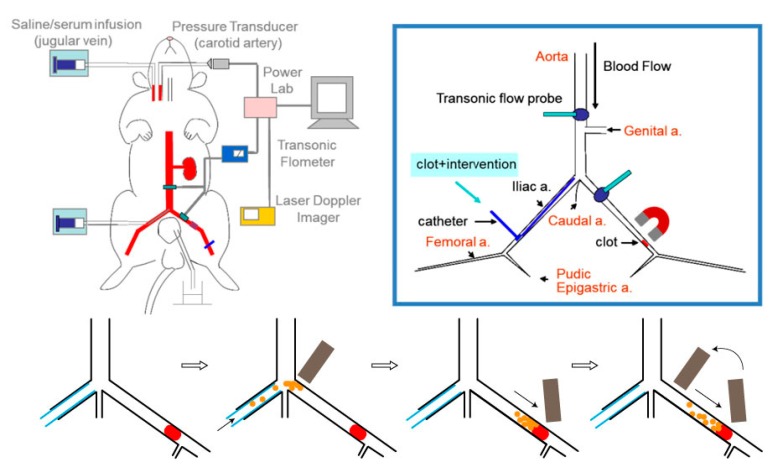
Schematic diagram of the rat iliac thromboembolic model and the magnetic guiding strategy. A preformed clot may be introduced from the right iliac cannula and lodged in the left iliac artery; FeCl_3_-induced thrombosis can be triggered in the left iliac artery. A mobile magnetic guiding strategy has been demonstrated crucial to achieve thrombolytic efficacy of rtPA nanocomposites administered from the right iliac artery (modified from [24]).

Application of magnetic field gradient may capture or manipulate MNPs, allowing “magnetic targeting” in drug delivery. In preclinical studies, simply using an NdFeB magnet may generate magnetic force enough to drag MNPs in circulation against hemodynamic forces in bigger artery with an emboli [24] or microvessels [25]. A magnetic field strength of 0.24 to 0.44 T has been used in animal models to induce magnetic targeting [24,26]. In a rat embolic model with preformed clot lodged in the iliac artery, magnetic guiding of MNPs caused local retention of MNPs at the target thrombus [24]. With a mobile NdFeB magnet at the site of thrombus, the nanocomposites could remain freely suspended in the blood (Figure 3), which is critical to allow interaction of the immobilized PAs with plasminogen in the thrombus, and subsequently induce thrombolysis [12,14]. In addition, application of magnetic force on the chitosan-coated MNPs encapsulating rtPA may induce agglomeration and preserve encapsulated rtPA from release, probably due to reduced surface area of the nanocomposties; removal of the magnet allows resuspension of the nanocomposites and serves as a signal to trigger release of rtPA [22]. Hu et al. reported using a custom-made rotational magnetic field (40 mT) above the head of mice after administration of rtPA incorporated in magnetic nanorods for treatment of a thrombus in the middle cerebral artery [26]. Although magnetic targeting appears feasible in some disease models, the application may be limited to the surface vessels, because of the limit of the permanent magnet. At the current time, there is no approved device for magnetic targeting that can be used in clinical setting.

### 3.2. Ligand Targeting

Development of nanocomposites with targeting ligand has been a very plausible strategy in development of thrombolytic therapeutics. Although rtPA is known to bind to fibrin, covalent conjugation of rtPA with anti-fibrin monoclonal antibody with 1000-fold higher affinity may enhance the thrombolytic efficacy of rtPA in a rabbit thrombosis model [27]. A variety of targeting ligands may enhance the binding of the nanocomposites to the thrombus, and thus enhance the thrombolysis efficacy, reduce the dose required, or reduce potential side effects. It is anticipated that glycoprotein IIbIIIa (GPIIbIIIa) [17,18,23] or P-selectin [23,28] on the surface of activated platelets may serve as a target in delivery. A fusion protein connecting antibody against GPIIbIIIa and single-chain urokinase plasminogen activator (uPA) was prepared and tested; however, the lysis effect is not different from that of commercial uPA in a mice thrombosis model [29]. Peptide containing sequential RGD amino acids [23] or cyclic RGD (cRGD) peptide [18] also exerts high affinity to glycoprotein IIbIIIa; these RGD-liposomes demonstrated significant targeting effects *in vivo*, as observed by microscopy. In addition, only 1/4 of the urokinase dose is required for cRGD-conjugated liposome to reduce the size of the thrombus [17].

In addition to the activated platelet, fibrin clot is another feasible target for drug delivery. Fibrin is the final product in the coagulation cascade, and the structure is further stabilized with activated clotting factor XIII (FXIIIa), which is a transglutaminase in freshly formed fibrin polymer. McCarthy et al., has demonstrated that nanocomposites with a FXIIIa-sensitive peptide shows an enhanced binding to thrombus with a similar efficacy as free rtPA *in vivo* [30]. In addition, peptide targeting fibrin-fibrinogen complex may be used to deliver micro-plasmin that directly degrades fibrin [31].

### 3.3. Shear Targeting

Korin et al. [32] developed shear-sensitive nanocomposites for delivery of rtPA based on observation that thrombosed vessel exhibits elevated fluid shear stress locally by one to two orders of magnitude. The rtPA preparation was prepared as microscale aggregates that break up into nanocomponents when exposed to high shear force in circulation. Such physical trigger has been proposed to be useful in target drug delivery for vasodilation, plaque stabilization and thrombolysis [33].

## 4. Assessment of *In Vitro* Thrombolysis

*In vitro* assays are often conducted under conditions remotely different from those *in vivo*, which contain physiological salts, protein components of the coagulation cascade, platelets and other blood cells that may participate in dynamic formation and lysis of the thrombus. Nevertheless, the enzyme activity of immobilized or encapsulated PAs has to be determined first *in vitro*, as part of the characterization of the nanocomposites. Such information is critical for calculation of the dose required for quantitative evaluation of the nanocomposites in animal models. Only those methods that are useful to generate results leading to a successful *in vivo* assessment are included.

### 4.1. Chromogenic Substrate Assay

A chromogenic substrate assay can be conducted to determine the amidolytic activity of plasminogen activator or plasmin. Amidolytic activity of PAs can be measured spectro-photometrically using the protease substrate S-2288, a specific chromogenic substrate for PAs, according to the manufacturer’s instruction, and presented as standardized activity units (IU). Activity retention after immobilization can be defined as the percentage of activity of immobilized PA compared with that of PA added initially. This method is simple with good reproducibility, and thus often used in optimization of the nanocomposites during preparation [11,12,14,21,22,26,30,34]. However, plasminogen is a much bigger molecule in structure, and therefore, interaction of plasminogen and PAs may not be as free as the small substrate.

### 4.2. Fibrin Degradation Assay

Fibrin zymography has been developed for determination of plasminogen-dependent fibrinolytic activity [35]. Fibrin clot lysis assay with fibrin-containing agarose plates has been used for *in vitro* analysis of the fibrinolytic activity [20]. Thrombolysis rate can be monitored with a commercial available analyzer by measurement of the meniscus movement in a cuvette with fibrin clot [15]. In addition, a commercial available ELISA kit for D-dimer can also be used for the same purpose [30].

### 4.3. Whole Blood Clot Lysis In Vitro

*In vitro* assay system using whole blood clot has been developed for determination of the *in vitro* dissolution or dissolution rate under static condition by measurement the hemoglobin in the supernatant at OD_405-415_ over time [14,21]. Alternatively, blood clot lysis induced by PAs vs. PA nanocomposites can be determined by a thrombolysis model driven by a constant pressure gradient at 37 °C [34], which is similar to the condition that maintains blood circulation *in vivo*. In this system, a collagen-coated glass capillary tube is vertically mounted below a reservoir and immersed in a PBS bath. Thrombin-induced whole blood clot can be produced at the bottom of the reservoir and the thrombolytic agents can be introduced above the blood clot in the reservoir which can be subjected to magnetic targeting by placement of a magnet next to the bottom of the reservoir and with rotation at 6 rpm. Blood generated in response to thrombolysis was allowed to drain from the reservoir, through the capillary, into the PBS bath. Time to blood flow was recorded as the time when blood first exited from the capillary into the PBS bath. However, such method requires tremendous manual intervention, and thus may be subjected to variation and potential artifact from the guiding strategy.

*In vitro* thrombolysis under dynamic condition can also be examined using microfluidic chips with two adjacent micro-channels connected by a 500 μM array of pillars, allowing infusion of thrombin solution to stimulate thrombus formation at the upper channel, while the lower channel was infused with whole blood [21]. To facilitate observation, fibrinogen-conjugated with Alexa 647 was used to label the clot in the microchannel of a flow chamber at a constant flow rate using a syringe pump; clot formation can be induced by perfusion of recalcified human blood through the channel surface covered with tissue factor and von Willebrand factor [15]. Two-dimensional areas of the clot can be visualized and recorded over time to calculate dissolution rate in response to thrombolytic nanocomposites.

### 4.4. Thromboelastometry

Thromboelastometry, or thromboelastography, originally described by Hartert in 1948 [36], is a technique demonstrating kinetics of clot formation and dissolution by measuring viscoelastic properties of the clot [37]. The reaction was initiated by addition of CaCl_2_ in buffer to a mixture of blood and PA preparation. Clot formation in the sample increased impedance of a pin on a rotating shaft (±4.75°), which was detected via reduced elasticity between the pin and a connected spring. The change of elasticity can be recorded for 2 h, allowing a full thromboelastogram assessment. The pattern of clot formation and lysis can be characterized as different parameters, including clotting time (CT, in seconds), alpha angle, clot formation time (in seconds), maximal clot firmness (MCF, in millimeter), actual clot firmness at 120 min (in millimeter), and lysis index (LI, % of MCF) at the indicated time etc.

This technique has been widely used for characterization of blood from patient and for assessment of the effects of drugs on clot production and stability [37,38], which provides a variety of parameters for characterization of blood coagulation, fibrin formation, and stability of the clot. This method has also been employed for analysis of the thrombolysis efficacy of nanocomposites [11,22], which provides non-biased assessment of effects on both coagulation and thrombolysis. Although concentration-dependent thrombolysis can be characterized, the range of the concentration has been shown to be quite narrow [22]. In addition, this method can also be used to determine whether the nanocarrier *per se* exerts an effect on coagulation or platelet activation, as an assessment of nanotoxicology.

## 5. Animal Models for Assessment of Thrombolytic Nanocomposites

Determination of the efficacy of the thrombolytic nanocomposites using thromboembolic animal models is required for translation. Characteristics of nanocomposites can be greatly altered in circulation due to the presence of salts and plasma proteins; formation of protein corona neutralizes the zeta potential of the nanocomposites and facilitates agglomeration [39]. In addition, endogenous inhibitors of plasminogen activators cause inactivation of the enzyme activity of PAs associated with the nanocomposites. Therefore, thrombolytic nanocomposites have to be examined not only *in vitro*, but also *in vivo*. During the past ten years, a variety of thromboembolic animal models have been used to determine the thrombolytic efficacy of these nanocomposites, as summarized in Table 1. In many cases, a thrombus was formed in situ by induction of endothelial damages by perivascular FeCl_3_. In other cases, a preformed clot was introduced to a designated site for easy assessment, depending on the methods for evaluation of the efficacy. In these thromboembolic models, the exact position of the clot may have to be identified in order to visualize using imaging technique or to conduct magnetic targeting. In case of nanocomposites with targeting ligands, endothelial-induced injury model in mice and a rat embolic model were used to demonstrate the targeting effects and thrombolytic effects, respectively [18,30].

### 5.1. Embolic Model by Introduction of a Preformed Clot

Rat embolic models were produced by introducing a preformed clot into the iliac artery [24] or carotid artery [18] for magnetic guiding and targeting. Introduction of the clot from the external carotid artery cannula with the internal carotid artery ligated allows the thrombus to locate in the common carotid artery, which creates a dead space that is convenient to retrieve the emboli for its weight after the treatment [18]. Introduction of the clot from the right iliac artery allows the clot lodging in the left iliac artery upstream to the pudic epigastric artery (Figure 3), with a suture placed around the iliac artery that can be stretched to reduce flow and manipulate clot lodging. After clot lodging, there is plenty of space for both magnetic guiding and placement of a flow probe between the bifurcation and the emboli. Placement of ultrasonic flow probes on the left iliac artery and the abdominal aorta allows measurements of blood flow changes in response to thrombolytic intervention, which is a functional assessment of thrombolysis. The flow measurement also assists in determination of the thrombus position. After introduction of the clot, the iliac flow reduces to near zero, indicating the clot is located upstream of the pudic epigastric artery, which is a requirement for the model for target thrombolysis. If the embolus is flushed further downstream, blood flow would be at least 2 mL/min, depending on how many vessel branches between the bifurcation and the emboli. Any thrombolytic agents administered would go through these arteries with less resistance, which is almost unlikely to perform magnetic targeting. Under the general anesthesia using a long-term thiobarbiturate, blood pressure of this model can maintain stable for hours, which is critical to determine flow changes as a functional assessment in response to intervention with thrombolytic nanocomposites after clot lodging. In target thrombolysis, a reduced dose of PA nanocomposites is anticipated to reach the same efficacy as that induced by a regular dose of free rtPA due to the targeting effects that increase the concentration of the drug at the target location. In fact, we have reported that different nanocomposites with 20% of regular rtPA doses may induce thrombolysis under magnetic targeting [11,12,14,22].

It is noteworthy that in testing thrombolysis induced by streptokinase nanocomposites, a rat embolic model was used with a human clot with plasminogen infusion, since streptokinase exerts species specificity that does not respond well on a rat clot [18]. Although never been tested, such strategy may also be useful, theoretically, when a targeting ligand exerts specie specificity to human targets.

### 5.2. Thrombosis Model Induced by Endothelial Damage

A piece of filter paper soaped with ferric chloride (FeCl_3_) solution and placed directly on the exposed blood vessels has been used to induce endothelial damage that stimulates thrombosis *in vivo*. The speed and extend of thrombus formation greatly depend on concentration of FeCl_3_, how long the filter paper gets in contact with the vessels, the artery size and animal species. It has been demonstrated that different arteries, including carotid and middle cerebral arteries [15,23,26,31], can be occluded by FeCl_3_-induced thrombosis. However, endothelial damage-generated robust signals continuously trigger thrombus formation, which is quite difficult to use thrombolytic agent alone to restore blood flow in this model [26,42]. In some situation, variation of the sensitivity of the thrombolytic treatment was observed [21], resulting in difficulties in reaching statistical significance.

To determine the animal model for testing the thrombolytic nanocomposites, we compared the FeCl_3_ thrombosis model with the rat embolic model described in the Section 5.1 on the same artery of the rats. A piece of filter paper (1 × 3 mm^2^; Toyo Roshi Kaisha, Ltd., Japan) soaped with FeCl_3_ (20%) was placed on the iliac artery of the rat for 3 min with the measurement of iliac flow and hind limb perfusion using ultrasonic flowmetry and laser speckle contrast imaging, respectively, as previously described in the rat embolic model [12,24]. Immobilized rtPA on polyacrylic acid-coated MNPs (MNP-rtPA; MNPs purchased from chemicell GmbH (Germany) was prepared as described in a previous study [12]. The results indicate that identical dose of rtPA nanocomposites as that used in the embolic model was not potent enough to induce thrombolysis; it requires co-administration of an anticoagulant heparin to induce thrombolysis and restore the iliac flow to the hind limb in this model (Figure 3). Although greatly increase in dose of rtPA may overcome the robust stimuli of thrombosis induced by injured endothelium, the FeCl_3_ model is much less sensitive than the embolic model for quantitative assessment of thrombolysis induced by nanocomposites, unless co-administration of an anticoagulant, such as heparin. In the previous studies using the FeCl_3_ model, thrombolysis treatment only induced a delay in occlusion, instead of recanalization [23,32]. Nevertheless, the FeCl_3_ model is still very useful in demonstrating the targeting effect of a specific ligand to the injured endothelium, fibrin clot or activated platelets.

Although hardly being tested in targeted delivery of PAs, laser induced vascular injury with [42] or without [43] the presence of a photo sensitizer in circulation may also trigger localized thrombosis in small vessels of the cremaster muscle, which can be more reproducible than that induced by FeCl_3_ due to less handling of the preparation. Kawata et al., employed balloon injury to induce endothelial injury and thrombotic occlusion, and the effects of nanocomposites was evaluated with transthoracic ultrasound [41]. In addition, needle or ultrasound-induced atherosclerotic plaque rupture also triggers thrombosis that appears to be a thrombosis model with pathological relevance [44].

## 6. Quantitative Measurement of Targeted Thrombolysis *In Vivo*

The methods employed for the efficacy study are dependent on how the thrombus is created and the size of the arteries. The following methods with advantages and disadvantages have been used for *in vivo* evaluation of thrombolytic nanocomposites as listed in Table 1.

### 6.1. Weight- or Radioactivity-Based Assessment

After thrombolytic treatment, the clot in a bigger artery can be removed and weighed, as a way for assessment of the efficacy of thrombolytic nanocomposites drugs [18,40]. Different strategies were employed to recover the remnant clot after treatment of the nanocomposites *in vivo*; nevertheless, recovery of the remaining clot can still be challenging due to loosened structure of the thrombus after treatment with PAs. Bi et al., inserted a piece of silk suture in a 25-cm polyethylene tubing connecting carotid artery and jugular vein, which served as a contact surface of thrombus formation and a supporting structure for clot collection from circulation [40]. Alternatively, a pocket of dead space in the artery can be created for placement and recovery of a pre-formed thrombus. Vaidya et al., placed a piece of clot made of human whole blood into the ligated internal carotid artery from a cannula in the external carotid artery of the rat [18]. However, nanocomposites may enter the clot and degrade the clot from inside, resulting in a loosened structure of clot that is prone to be broken in handling, and thus causing variation and even overestimating the effects of thrombolytic treatment.

Alternatively, introduction of a premade ^125^I-fibrin to the jugular vein of mice to produce an embolic model [30] may serve a more reliable way for assessment of the thrombolytic treatment. The radioactive fibrin clot was prepared by mixing human plasma with trace amount of ^125^I-fibrinogen to produce the thrombus for embolization to rat supplemented with human plasminogen and heparin. At the end of the experiments, both hearts and lungs were dissected and subjected to gamma counting to quantitate the remaining clots. However, most investigators prefer to minimize handling such materials that generate costly radioactive hazard, especially in the whole animal study.

### 6.2. Imaging Assessment In Vivo

Different imaging techniques can be employed to evaluate thrombolysis in response to drug intervention. Although histology study may be used to provide evidence showing thrombolysis in the vessel, the one-time point observation is not convincing to reach the conclusion, and other evidence has to be provided [31]. Fluorescent microscope was used to observe the binding of FITC labeled nanomocomposites with cRGD as the targeting ligand in the FeCl_3_ thrombosis model [17].

Fluorescence labeling of platelets and leukocytes with rhodamine 6G prior to induction of endothelial damage allows visualization of the thrombus formation and dissolution with microscopy, and the signal can be quantitated for analysis [17]. Although thrombus localization of fluorophore-labeled nanocomposites may be visualized with microscopy, other methods are often considered to be more reliable for quantitation of the thrombolysis effects [18], probably because that imaging assessment can be more subjected to bias and variation.

### 6.3. Functional Assessment In Vivo

**Figure 4:**
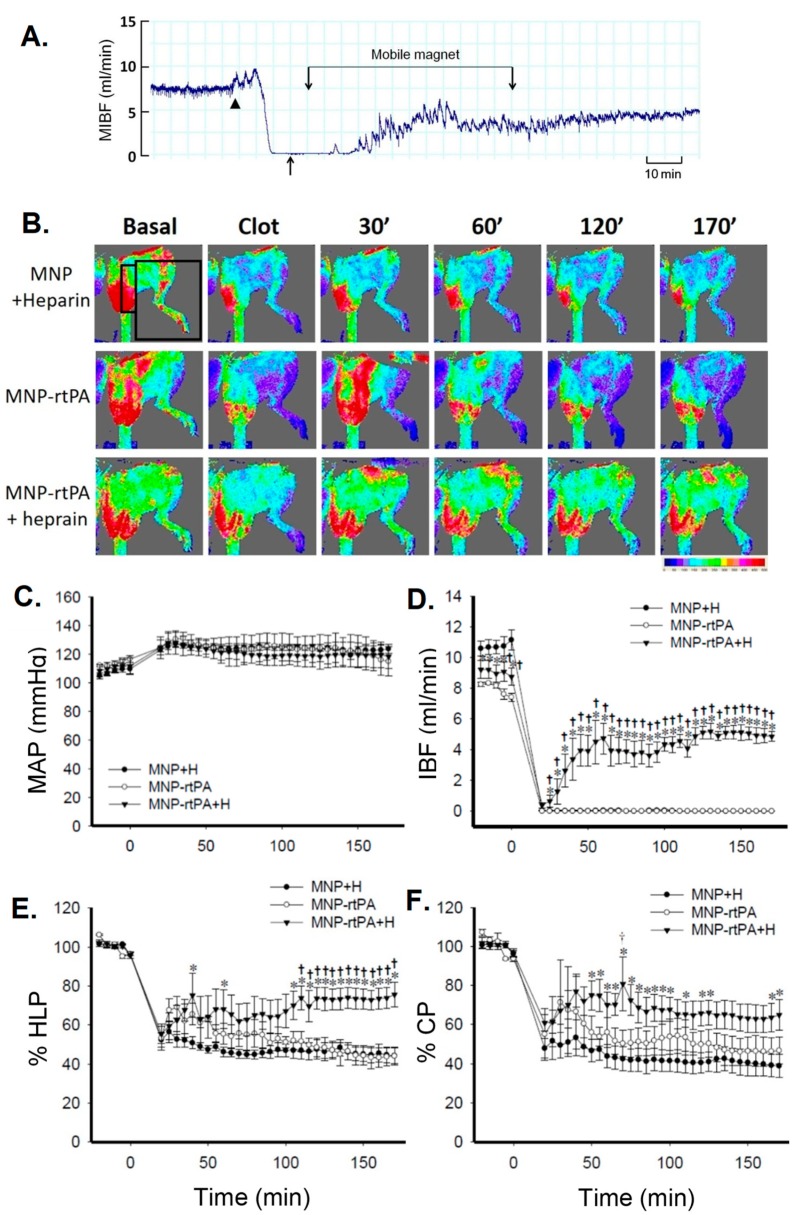
Target thrombolysis induced by immobilized rtPA plus heparin under magnetic guiding in a FeCl_3_ thrombosis model of the rat. (**A**) illustrates representative effects of MNP-rtPA plus heparin on the mean iliac artery blood flow (MIBF). FeCl_3_-induced thrombosis (arrow head) reduced left iliac blood flow to below 1 mL/min; MNP-rtPA (0.2 mg/kg) with heparin (500 mg/kg plus 500 IU/kg^.^hr for one hr) was administered from the right iliac artery, as indicated by the arrow. (**B**) Tissue perfusion of hind limb (HLP) and cremaster muscle (CP) was measured by laser speckle contrast imaging; the signals acquired in the areas are denoted as in the squares. Mean arterial pressure (MAP; **C**), mean iliac blood flow (MIBF; **D**), HLP (**E**) and CP (**F**) were measured with time. FeCl_3_ (20%) filter paper was placed on the left iliac artery at time 0. MNP-rtPA with heparin (H; 500 mg/kg plus 500 IU/kg^.^h for one hour; n = 5) or equivalent MNP with heparin was administered from the right iliac artery 5 min after complete occlusion. Values were presented as mean ± SE. *, *p* < 0.05 compared with the corresponding control group. †, *p* < 0.05 compared with the corresponding MNP-rtPA group.

Thrombus-induced blood flow reduction is the major cause of thromboembolism-induced pathological consequence, such as ischemia and tissue necrosis. Therefore, direct measurement of flow restore of the vasculature after thrombolytic therapy may serve as a marker for functional restore [11,12,14,22,26]. Measurement of hemodynamic changes before and after drug intervention can be conducted real-time using ultrasonic or laser Doppler flowmetry (Figure 3), which acquire absolute volume flow and relative changes of tissue perfusion, respectively. In Figure 4A,D, the MIBF was recorded with time using an ultrasonic flow probe placed on the iliac artery with the emboli. In addition, laser speckle contrast imaging was used for assessment of the red blood cell movement/speed in the vessels, as assessment of tissue perfusion. In Figure 4B, two-dimensional speckle imaging illustrates the pattern of tissue perfusion that changes correlated with the drug intervention, which can also be quantitated as a function of time (Figure 4E,F). Alternatively, measurement of pulmonary arterial pressure may serve as functional assessment in animal models with pulmonary embolism. It has been shown that an increase in pulmonary arterial pressure associated with pulmonary embolism was reduced by a shear-sensitive rtPA preparation in mice [32]. For a stroke model with thrombus in the cerebral artery, quantitation of the infarct size of the brain using 2,3,5-triphenyltetrazolium hydrochloride staining 24 h after induction of ischemia can be used as a functional assessment with clinical relevance [26].

### 6.4. Pharmacological Consideration in Animal Study

To facilitate translation, “Animal Research Reporting Experiments (ARRIVE) guidelines” have to be considered. Starting 2016, inplementing ARRIVE guidlines on reporting experimental results from animal study is required by British Journal of Pharmacology [45] and other journals. It is suggested by the guideline that the description of the methods has to be transparent and in sufficinet details to permit independent repetition and interpretation of the results. In addition, “optimized”, rather than “smallest” number of animals shall be used, since underpowered studies are useless and wasted. Data subjected to statistical analysis at replicates, of at least 5. Determination of the outliers within the database has to be described, which has been hardly mentioned in nanomedicine-related areas. The analysis should have a group size (n), no guidelines also emphasize animal wellfae, for experimental results from healthy animals are more likely to be reproduced.

Although pharmacological efficacy of PA nanocomposites has been demonstrated in many studies using different animal models, few studies evaluate the potential toxicity of these nanocomposites. It has been demonstrated that cytotoxicity in human endothelial cells in culture was not significant [17]. In addition, tail bleeding time is often acquired as an indicator for systemic toxicity. It has been demonstrated that cRGD-liposomal preparation of urokinase exert much smaller effect on the bleeding time than that of the free drug [17]; whereas urokinase-conjugated MNPs-induced increase in bleeding time was significantly attenuated with application of magnetic field [40]. A recent study suggested that a porous magnetic iron oxide microrods incorporated with rtPA exerted no significant impact on hepatic or renal function, as evaluated with plasma biomarkers [26].

## 7. Conclusions

In addition to target treatment of tumor, target thrombolysis has attracted tremendous attention in the area of nanomedicine in recent years. Unlike most of tumors, thrombus is located in circulation, thus parenteral administration of the thrombolytic agent allows its reaching the pathological target without layers of barriers. The challenge is the potentially complicated thrombus formation in size and location that may hinder achieving functional restore and therapeutic outcome in a predictable and reproducible manner required for evaluation of the efficacy. Therefore, conducting the experiments based on ARRIVE guidelines using a well characterized animal model with an appropriate evaluation method can be a crucial step paving the way in development and translation of nanocomposites for target thrombolysis

## 8. Future Perspectives

All the targeting strategies may readily alter the pattern of biodistribution and pharmacokinetics of the nanocomposite, and thus increase its therapeutic efficacy. Although magnetic targeting may not be easily applied in clinical setting, superparamagnetic characteristics still allow MNPs to be preferable in development of drug carriers. For target thrombolysis, ideal nanocomposites are composed of encapsulated or protected PAs with ligand/shear targeting capacity that allows administration of the nanocomposites from a remote site, such as vein, to reach a clot deep in the tissue and release the payload at an appropriate rate.

## Figures and Tables

**Table 1:** Animal models used for evaluation of nanocomposite-induced target thrombolysis with statistical significance.

Models	Thrombus Location	Targeting Strategy Ligand: Target	*In Vivo* Thrombolysis Assessment	Species (n)	Ref
Thrombosis induced by FeCl_3_	mesenteric vessel	cRGD: GPIIbIIIa	imaging: thrombus size	mice (5)	[17] ^1^
	mesenteric vessel	---	imaging	mice (≥10)	[21]
	mesenteric vessel	Fucoidan: P-selectin	imaging	mice (32)	[28]
	mesenteric vessel	Shear force	imaging: occlusion time	mice (N/A)	[32]
	carotid a.	Multivalent peptide:GPIIbIIIa/P-selectin	imaging: occlusion time	mice (4)	[23]
	carotid a.	Magnetic targeting	imaging fluorescent fibrin: time to reperfusion	rats (5) & rabbits (5)	[15]
	carotid a.	Peptide: fibrin-fibronectin complex	histology	mice (4–6)	[31]
	middle cerebral a.	Magnetic targeting	flowmetry & infarction	mice (4–5)	[26] ^1^
	vena cava	Peptide: GPIIbIIIa	wet weight of clot	rats (5–6)	[20]
Thrombosis induced by thrombin	jugular v.	Glu-plasminogen:	^125^I-fibrin radioactivity	rabbits (5–8)	[16] ^1^
		fibrin			
	abdominal aorta	Ultrasound	flowmetry	rabbits (4–5)	[19]
	vena cava	Peptide: fibrin-fibronectin complex	wet weight of clot	rats (10)	[31]
Arteriovenous shunt thrombosis model	A-V tubing with silk suture	Magnetic targeting	wet weight of clot	rats (10–11)	[40]
Balloon injury model	coronary a.	Ultrasound	ultrasound &ventricular ejection fraction	swine (10)	[41]
Embolic model	iliac a.	Magnetic targeting	flowmetry	rats (6–7)	[12,14] ^1^
	iliac a.	Magnetic targeting & controlled release	flowmetry	rats (8–9)	[22] ^1^
	iliac a.	Magnetic/thermal controlled release	flowmetry	rats (5–10)	[11] ^1^
	carotid a.	cRGD: GPIIbIIIa	wet weight of clot	rats (6)	[18]
	pulmonary a.	Shear force	pulmonary a. pressure	mice (N/A)	[32] ^1^
	pulmonary a.	Peptide: FXIII	^125^I-fibrin radioactivity	mice (9–11)	[30]
	right ventricle				

^1^ demonstrating lower dose of PAs in nanocomposites required to induce thrombolysis. N/A, not available.
